# Supplementary material for: Mapping the evolution of fertility support policies in China: A content and instrumental analysis
Source: PLoS One. 2025 Oct 9;20(10):e0332137. doi: 10.1371/journal.pone.0332137 (PMC12510515; doi:10.1371/journal.pone.0332137)
Supplement: S1 Appendix — (ZIP) [file pone.0332137.s001.zip › S1 Appendix. 226 original policy documents/77-国务院关于设立3岁以下婴幼儿照护个人所得税专项附加扣除的通知(FBM-CLI-2-5115421).doc]

国务院关于设立3岁以下婴幼儿照护个人所得税专项附加扣除的通知

**发布部门：** [国务院](https://www.pkulaw.com/law/chl?Aggs.IssueDepartment=5)

**发文字号：**国发〔2022〕8号

**发布日期：**2022.03.19

**实施日期：**2022.01.01

**时效性：** [现行有效](https://www.pkulaw.com/law/chl?Aggs.TimelinessDic=01)

**效力级别：** [国务院规范性文件](https://www.pkulaw.com/law/chl?Aggs.EffectivenessDic=XC0203)

**法规类别：** [个人所得税](https://www.pkulaw.com/law/chl?Aggs.Category=0320305)

国务院关于设立3岁以下婴幼儿照护个人所得税专项附加扣除的通知
（国发〔2022〕8号）

各省、自治区、直辖市人民政府，国务院各部委、各直属机构：
　　为贯彻落实《中共中央 国务院关于[优化生育政策促进人口长期均衡发展的决定](https://www.pkulaw.com/chl/1247bc81dccaeed3bdfb.html)》，依据《[中华人民共和国个人所得税法](https://www.pkulaw.com/chl/d74fa423af05cffdbdfb.html)》有关规定，国务院决定，设立3岁以下婴幼儿照护个人所得税专项附加扣除。现将有关事项通知如下：
　　**一、**纳税人照护3岁以下婴幼儿子女的相关支出，按照每个婴幼儿每月1000元的标准定额扣除。

　　**二、**父母可以选择由其中一方按扣除标准的100%扣除，也可以选择由双方分别按扣除标准的50%扣除，具体扣除方式在一个纳税年度内不能变更。

　　**三、**3岁以下婴幼儿照护个人所得税专项附加扣除涉及的保障措施和其他事项，参照《个人所得税专项附加扣除暂行办法》有关规定执行。

　　**四、**3岁以下婴幼儿照护个人所得税专项附加扣除自2022年1月1日起实施。

国务院
　　2022年3月19日

© 北大法宝：（[www.pkulaw.com](https://www.pkulaw.com/)) 专业提供法律信息、法学知识和法律软件领域各类解决方案。北大法宝为您提供丰富的参考资料，正式引用法规条文时请与标准文本核对。
欢迎查看所有[产品和服务](https://www.pkulaw.com/helps/2.html)。法宝快讯：[如何快速找到您需要的检索结果？](https://www.pkulaw.com/helps/70.html)[法宝](https://www.pkulaw.com/helps/69.html)V6有何新特色？

扫描二维码阅读原文

**原文链接：**<https://www.pkulaw.com/chl/fc36eb215768159abdfb.html>
